# Proteoglycans in Articular Cartilage and Their Contribution to Chondral Injury and Repair Mechanisms

**DOI:** 10.3390/ijms241310824

**Published:** 2023-06-28

**Authors:** Lourdes Alcaide-Ruggiero, Ramón Cugat, Juan Manuel Domínguez

**Affiliations:** 1 Departamento de Medicina y Cirugía Animal, Facultad de Veterinaria, Universidad de Córdoba, Hospital Clínico Veterinario, Campus de Rabanales, Ctra. Madrid-Cádiz Km 396, 14014 Córdoba, Spain; 2 Fundación García-Cugat, Plaza Alfonso Comín 5–7, 08023 Barcelona, Spain; 3 Instituto Cugat y Mutualidad de Futbolistas Españoles, Delegación Catalana, 08023 Barcelona, Spain

**Keywords:** proteoglycans, extracellular matrix, articular cartilage, chondral injury, biomarkers

## Abstract

Proteoglycans are vital components of the extracellular matrix in articular cartilage, providing biomechanical properties crucial for its proper functioning. They are key players in chondral diseases, specifically in the degradation of the extracellular matrix. Evaluating proteoglycan molecules can serve as a biomarker for joint degradation in osteoarthritis patients, as well as assessing the quality of repaired tissue following different treatment strategies for chondral injuries. Despite ongoing research, understanding osteoarthritis and cartilage repair remains unclear, making the identification of key molecules essential for early diagnosis and effective treatment. This review offers an overview of proteoglycans as primary molecules in articular cartilage. It describes the various types of proteoglycans present in both healthy and damaged cartilage, highlighting their roles. Additionally, the review emphasizes the importance of assessing proteoglycans to evaluate the quality of repaired articular tissue. It concludes by providing a visual and narrative description of aggrecan distribution and presence in healthy cartilage. Proteoglycans, such as aggrecan, biglycan, decorin, perlecan, and versican, significantly contribute to maintaining the health of articular cartilage and the cartilage repair process. Therefore, studying these proteoglycans is vital for early diagnosis, evaluating the quality of repaired cartilage, and assessing treatment effectiveness.

## 1. Introduction

The extracellular matrix (ECM) is widely acknowledged within the scientific community as the most complex structural organization present in an organism [1]. Composed of an intricate and finely organized network of collagens, proteoglycans (PGs), fibronectin, laminins, elastin, glycosaminoglycans (GAGs), and glycoproteins, the ECM serves as a vital support system for cells, tissues, and organs, imparting them with their unique mechanical and chemical properties [2,3]. This network, along with other proteins and growth factors [4], gives each tissue its chemical and mechanical properties [5]. The morphological integrity of articular cartilage ECM mainly determines the proper functioning of the joint [6]. The ECM of articular cartilage can be classified into three regions: The PCM, the territorial matrix, and the interterritorial matrix [7,8]. The PCM is the layer closest to the cell, while the territorial matrix surrounds the cell with a layer of fibrillar collagen. The interterritorial matrix is the largest region and contains most of the material located outside of the cells in cartilage [9]. The unique structure of the PCM allows for chondrocyte–matrix interactions, thereby regulating chondrocyte phenotype and cell survival [10]. The PCM is rich in PGs, collagens, basement membrane proteins, and non-collagenous glycoproteins, which interact and form a mesh-like structure [7,11,12]. Furthermore, the PCM is connected with the neighboring tissue through a meshwork of fine collagen fibrils, PGs, and fibronectin, which together create the territorial matrix [9].

Articular cartilage is a connective tissue that hides the surface of bones where they meet in joints. This cartilage is composed of chondrocytes (between 1% and 5% of the cartilage volume) embedded within an organized ECM of collagen, PGs, and other proteins (approximately 65%, 15%, and 15%, respectively) [13]. It is endowed with a specialized structure that confers upon it the necessary biomechanical characteristics to withstand joint loading. Specifically, this structure imparts compressive strength to the cartilage and ensures maintenance of fluid and electrolyte balance, both of which are critical for the optimal functioning of the joint [14]. This structure has limited reparative and regenerative capabilities due to its lack of vascularity and the fact that mature chondrocytes lose their ability to migrate, proliferate, and synthesize their surrounding matrix [15,16]. Cartilage serves two key functions in the body providing near-frictionless movement between bones and counteracting the compressive forces that are exerted across the joint during movement. These functions are largely attributable to the presence of PGs within the ECM of cartilage [17]. 

Chondral injuries in patients with healthy cartilage generally have a traumatic origin and can evolve with cartilage degeneration and osteoarthritis (OA) [18]. OA is the primary cause of disability in adults in the United States [19], with the United Nations projecting that the number of people suffering from OA will reach 130 million by 2050 [20]. Presently, OA affects more than 10% of the elderly population [19]. A defining characteristic of OA is the gradual deterioration of articular cartilage due to the permanent degradation of the cartilage ECM and the remodeling of adjacent joint tissues. This degeneration leads to joint dysfunction, limited mobility, and excruciating pain during routine activities [21,22]. 

Despite extensive research efforts aimed at elucidating the molecular mechanisms responsible for the onset and progression of OA, as well as intrinsic cartilage repair processes, many aspects of these processes remain unclear [23]. Determining the key molecules involved in these processes would enable early diagnosis and preventive treatment of OA, ideally leading to a 100% effective solution to treat chondral lesions. For these reasons, the present review provides an overview of PGs as one of the main molecules of articular cartilage, describing the PGs present in this particular tissue and the role they play in both healthy and damaged cartilage. Additionally, this review highlights the importance of including PGs assessment to evaluate the quality of repaired articular tissue. Furthermore, a visual and narrative description of the distribution and presence of aggrecan in healthy cartilage is provided.

## 2. General Characteristics of Proteoglycans

PGs are a class of intricate macromolecules that can be found in various forms within most tissue [24]. These molecules consist of a central protein that has different quantities of GAG side chains linked to it. PGs are categorized into multiple families based on their occurrence in cells and tissues, their interactions with other macromolecular constituents, and the particular structures of their core proteins [25]. In this way, PGs correspond to a wide variety of functions, such as structural functions, regulation of enzymatic activity, and cell surface receptors. During development and tissue repair, PGs play an important role in regulating gradients and the availability of growth factors, cytokines, chemokines, and morphogens [26]. 

PGs have been a subject of study since the 20th century, but it was not until the late 1960s, when Sajdera and Hascall developed an innovative extraction protocol, that PGs gained recognition [27]. Throughout the 1970s, a considerable amount of research was published on the isolation, purification, and characterization of PGs from ECM in healthy and pathological conditions. During this time, studies primarily focused on tissue with a high PG content, such as cartilage, the aorta, and skin, due to methodological limitations. With the advent of molecular biology techniques, research on PGs has become more accessible, leading to new insights into their structure and function [24].

### 2.1. Structure

**Figure 1:**
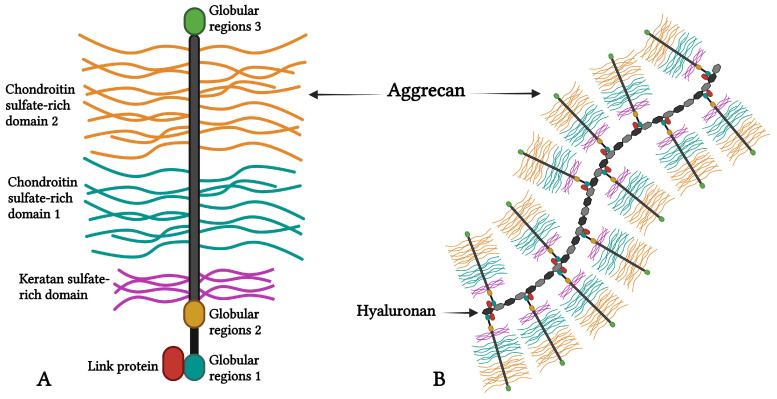
Aggrecan and PG aggregates structure. (**A**) Aggrecan, a hybrid PG consisting of a protein core with three lobular regions, keratan sulfate, and chondroitin sulfate. (**B**) PG aggregates composed of a central hyaluronan filament with numerous aggrecan molecules bound together. Modified from Roughley et al. [17].

GAGs are covalently attached to the core protein of the PG. They are unbranched and often resemble long polysaccharides with a repeating disaccharide structure [26]. In most PGs, GAGs comprise more than 50% of the total molecular mass and mediate biological functions. The molecular composition of the GAG chains determines the classification of PGs as chondroitin sulfate PGs (CSPGs), heparan sulfate PGs (HSPGs), keratan sulfate PGs (KSPGs), or dermatan sulfate PGs (DSPGs). Some PGs are considered hybrid PGs since they contain multiple types of chains. For instance, aggrecan is the predominant PG in cartilage, consisting of a core protein with three disulfide-linked globular regions (G1, G2, and G3) and an intervening extension region. CSPG is covalently linked from the G3 domain to over half of the protein core, but it also contains KSPG near the G2 domain (Figure 1A) [17,26].

### 2.2. Biosynthesis

The protein core of PGs is synthesized by ribosomes and transported to the rough endoplasmic reticulum. Their glycosylation takes place in the Golgi apparatus in multiple enzymatic stages, requiring different glycosyltransferases. A specialized link of tetrasaccharide is attached to the core protein’s serine side chain to initiate polysaccharide growth. The PG is subsequently transported to the ECM of the tissue through secretory vesicles. The production of core proteins and carbohydrate chains can be performed independently, owing to the intricate nature of the macromolecules. Glycosylation, a process that necessitates a significant amount of energy, especially in the case of aggrecan, involves the use of several enzymes in excess. Numerous hydrolases are involved in carbohydrate degradation, either extracellularly or intracellularly in lysosomes [26].

### 2.3. Classification

The classification of PGs is a complex process. It involves grouping nearly all known PGs of the mammalian genome into four major classes based on factors, such as their cellular localization, similarity in gene/protein structure, and the specific protein units found within their protein cores. PGs can be classified as intracellular PGs, cell surface PGs, pericellular PGs, and extracellular PGs (Table 1) [28].

Serglycin is the only intracellular PG, usually with heparin side chains. Serglycin is a PG present in the granules of mast cells and functions as a binding agent for most of the intracellular proteases that are stored in these granules [29]. This PG is expressed by all inflammatory cells and is stored within intracytoplasmic granules, where it interacts with and regulates the activity of various inflammatory mediators, chemokines, cytokines, and growth factors [30]. Furthermore, serglycin has also been detected in several non-immune cell types, including chondrocytes, endothelial cells, and smooth muscle cells [31].

Cell surface PGs comprise thirteen genes, of which seven are responsible for encoding transmembrane PGs and the other six for glycosyl-phosphatidyl-inositol (GPI)-anchored PGs. All PGs belonging to this group contain heparan sulfate side chains, with the exception of NG2 and phosphacan [28].

Pericellular and basement membrane region PGs consist of four PGs that are closely linked to the surfaces of many types of cells through integrins and other receptors. However, they can also act as components of most basement membranes. Pericellular PGs are mostly HSPGs and include perlecan and agrin, which will be discussed in this review [28].

Extracellular PGs are the largest group with twenty-five different genes, divided into three subgroups. The first subgroup contains four genes that encode hyalectans, including aggrecan, versican, neurocan, and brevican, which are key structural components of cartilage, blood vessels, and nervous systems. The second subgroup includes eighteen small leucine-rich PGs (SLRPs), which perform various functions and signal through different receptors. The third subgroup consists of three testicans, which are calcium-binding HSPGs [28].

## 3. Extracellular Matrix Proteoglycans in Articular Cartilage

Aggrecan and versican form large aggregates that are critical for maintaining the pericellular environment around the cell. However, the level of versican decreases with age [7,32]. Apart from large aggregating PGs, non-aggregating PGs, such as biglycan, decorin, versican, and perlecan are also present [7]. Aggrecan is the most abundant PG in terms of weight in articular cartilage, however, in young cartilage, similar amounts of aggrecan, biglycan, and decorin are present on a molecular level [24].

### 3.1. Aggrecan 

Aggrecan is a predominant PG found in typical hyaline cartilage, such as articular cartilage [33], where it exists as PG aggregates [34]. These aggregates consist of a central filament of hyaluronan, to which several aggrecan molecules are attached. The filament of hyaluronan has a protein core of approximately 200 kDa molecular mass, to which chondroitin sulfate, keratan sulfate, and 50 N- and O-linked oligosaccharide chains are attached (Figure 1B) [17,33]. Each PG aggregate can contain over 1000 aggrecan molecules [33]. Aggrecan is responsible for providing the viscoelastic properties of cartilage and plays an important role in cell–ECM interaction, binding, and the release of growth factors and morphogens [35]. 

When exposed to water, the sulfated GAG chains of aggrecan become hydrated, leading to swelling and the expansion of its molecular domains [17,36]. However, in the ECM, the swelling is mitigated by collagen fibrils that provide the structural support for cartilage. In the presence of an adequate quantity of aggrecan, a state of equilibrium is attained in which the swelling of aggrecan is balanced by the tensile forces generated by stretching the collagen fibrils. To ensure optimal cartilage function, it is necessary to have high concentrations of aggrecan to achieve this balance [17]. This mechanism is the foundation for the hydrodynamic viscoelastic properties of articular cartilage [36].

**Figure 2:**
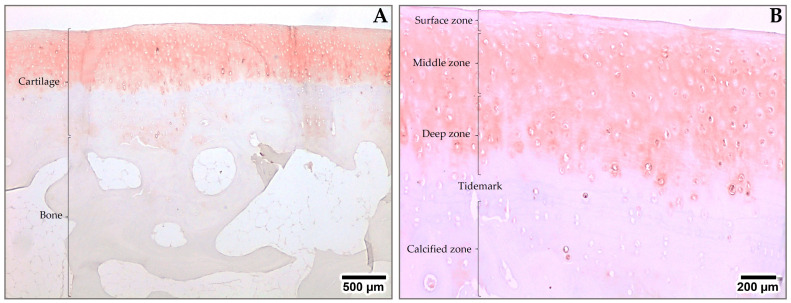
Immunohistochemical staining of aggrecan in healthy sheep articular cartilage [38]. (**A**) Lower magnification image depicting the cartilage and underlying bone. (**B**) Higher magnification image revealing intricate details of all cartilage areas. The adivin-biotin-complex method was used for the immunohistochemistry. Enzymatic pre-treatment with hyaluronidase was used. The primary antibody used was anti-aggrecan (ab3778, Abcam, Cambride, UK) at 1/100 dilution in PBS containing 10% normal goat serum.

Although aggrecan is an essential functional element in articular cartilage and plays an important role in OA and chondral repair processes, it is difficult to find accurate information and images of aggrecan’s distribution within articular cartilage. For this reason, samples of healthy sheep articular cartilage from a previous study [37] were used to describe the distribution of aggrecan via immunohistochemistry (Figure 2). We observed aggrecan to be present in the cartilage zone, while it was completely absent in bone tissue (Figure 2A). When aggrecan´s presence was analyzed by zones, this PG was highly abundant in the upper zones of the cartilage (superficial, middle, and deep zones), but almost absent in the calcified zone. In the upper zones, aggrecan was found in the territorial and inter-territorial matrix, in the pericellular matrix (PCM), and in chondrocytes. The aggrecan presence decreased when the tidemark was approached. In the calcified zone, this PG was only observed in some chondrocytes and their PCMs (Figure 2B).

### 3.2. Biglycan 

Biglycan and decorin belong to SLPRs family of PGs and are characterized by their small size and abundance of leucine repeats. They are composed of a core protein that has a molecular mass of about 40 kDa, to which dermatan sulfate chains are attached [39,40]. In cartilage, biglycan is one of the small PGs present in the ECM, binding to other molecules and helping in stabilizing the matrix [13]. In particular, this PG is located in the PCM [41]. 

Biglycan can interact with bone morphogenetic protein, which plays an important role in the metabolism of cartilage and bone [24]. During skeletal development, biglycan is present in a rim of chondrocytes close to the articular surface [42]. Furthermore, a study conducted by Han et al. [41] demonstrated that biglycan does not have a significant role in regulating cartilage degradation. However, biglycan has a strong function in the structure of subchondral bone.

Biglycan undergoes proteolytic processing as an individual ages, leading to the removal of the amino-terminal region that carries dermatan sulfate chains. Consequently, biglycan without glycanation tends to accumulate in the cartilage matrix over time [43].

### 3.3. Decorin 

Decorin, along with biglycan, is the most abundant small PG present in the articular cartilage ECM. Decorin is distributed in the pericellular, territorial, and interterritorial matrixes of cartilage [6,41] and has an important role in maintaining the cartilage’s structural integrity [41]. It functions as a “physical linker” that regulates the assembly of the aggrecan network in the ECM of the cartilage [6]. Moreover, decorin can also bind to the transforming growth factor beta and sequester it in the matrix [24]. This PG remains within intact articular cartilage at all ages [43].

### 3.4. Versican 

In the early stages of chondrocyte differentiation, versican is transiently expressed and incorporated into the ECM, but disappears as it is replaced by aggrecan [44]. A previous study showed that mice lacking versican expression achieved endochondral ossification, indicating that versican was not crucial for cartilage development [45]. 

### 3.5. Perlecan 

The cartilage ECM contains perlecan, whose presence in articular cartilage is unexpected since this PG is typically associated with basement membranes, which are absent in cartilage [43]. Perlecan is a multifunctional PG that promotes the proliferation, differentiation, and matrix synthesis of chondrocytes through its interactions with growth factors, morphogens, and ECM-stabilizing glycoproteins [46,47]. It also contributes to the mechanosensory properties of cartilage through pericellular interactions with fibrillin, type IV, V, VI, and XI collagen, and elastin [48]. These interactions help in stabilizing and enhancing the functional properties of mature cartilaginous ECM [49]. Perlecan plays a role in the maturation of chondroprogenitor stem cells and the development of pluripotent migratory stem cell lineages that contribute to joint formation and early cartilage development [50]. 

### 3.6. Proteoglycan Interaction with Other Molecules in the Extracellular Matrix of Articular Cartilage

The PCM, located closest to the chondrocytes, contains type VI collagen, which forms a microfibrillar network anchoring the chondrocyte to the ECM [8]. The PCM binds to type II collagen, aggrecan, and hyaluronan, and interacts with biglycan, decorin, and type IX collagen [11,51]. Type VI collagen is related to the PCM PGs, and biglycan and decorin are essential for the structural integrity of the PCM by connecting it to the territorial/interterritorial matrix. They also serve as functional bridges between type II and VI collagen [11]. Perlecan, along with type VI collagen, contribute to the organization and mechanical stability of the PCM, and affect its modulus [49]. Col6a1 inactivation results in a reduction in genes encoding aggrecan, biglycan, and decorin, which are important during chondrogenesis [52]. When bound to type VI collagen, perlecan has cytoprotective properties [53]. 

## 4. Role of Proteoglycans in Chondral Injuries

One pathological feature of chondral repair limitations, such OA, is the depletion of matrix macromolecules from cartilage, particularly PGs [54]. The biosynthesis and degradation of cartilage PGs entail multiple enzymes, and there is evidence suggesting that deficiency or disruption of any of these enzymes can lead to severe cellular or organ dysfunction or damage. The production of deficient PGs can impact their charge density or interactions with other extracellular components, altering the structure and properties of the cartilage. The insufficient degradation of PGs can lead to a limited accumulation of degradation products that can have deleterious effects on organisms [24]. 

The loss of PGs in the ECM increases hydraulic permeability and decreases solid charge density, thereby reducing the cartilage´s ability to adequately support mechanical loads [55,56].

### 4.1. Aggrecan 

The event that triggers the depletion of aggrecan in cartilage could be caused by trauma, inflammation, or excessive loading of the joint. Once damage has begun, and the endogenous repair capacity of articular cartilage is low, further damage, such as OA, may be an inevitable effect [17].

Aggrecan content and composition appear to be strongly related to tissue status. In aging cartilage, there is a reduction in the overall amount of aggrecan [24]. In the OA joint, catabolic processes destroy both the hyaluronan backbone of the aggregate and the core protein of the aggrecan molecules, thus impairing their function and making articular cartilage susceptible to erosion. Aggrecan degradation is caused by proteinases, hyaluronidases, and free radicals [17]. Some in vivo studies have indicated that levels of aggrecan are initially high in the early stages of OA to prevent cartilage loss. However, over time, the levels of aggrecan decrease due to proteolytic activity, which ultimately leads to the destruction of cartilage [57,58]. 

Proteolytic cleavage of aggrecan produces two fragments, one of which remains attached to hyaluronan, while the other fragment loses its interaction with hyaluronan and can easily diffuse through the ECM and become lost in the synovial fluid [55,56,57]. The fragments that remain bound to hyaluronan can persist in the tissue for many years, hampering the repair process and occupying space that could be used for binding newly synthesized aggrecan [17]. Aggrecan fragments that appear in the synovial fluid can serve as biomarkers for cartilage degradation, with higher concentrations indicating increased degradation in patients with OA [17,59]. Normal articular cartilage necessitates a high concentration of aggrecan, a high degree of sulfation, and the capacity to form large aggregates, all of which are compromised in OA joints [17].

### 4.2. Biglycan and Decorin 

In human cartilage, decorin and biglycan are not typically found on the surface of articular cartilage, but their levels increase in the deeper regions of the tissue [60]. In cases of OA, the levels of these PGs are significantly upregulated, which is believed to be a compensatory mechanism by chondrocytes to counteract cartilage degeneration [61]. This is supported by the finding that decorin acts as a “physical linker” to enhance the molecular association of aggrecan, which in turn increases the structural integrity of aggrecan networks in healthy cartilage ECM and reduces the loss of fragmented aggrecan from degenerative cartilage [41].

At present, it is acknowledged that both SLRPs play a crucial role in cartilage function and pathology. Nevertheless, it is still unclear how decorin and biglycan function individually or in conjunction to control the onset and advancement of OA [41]. A recent study reported that decorin insufficiency leads to altered ECM biomechanical characteristic and cartilage stiffness [62].

After tissue injury, biglycan and decorin can be released in a soluble form from the cartilage matrix, which could act as an endogenous warning signal [63,64].

### 4.3. Perlecan 

Perlecan is a molecule that possesses properties that are important for cartilage repair, including chondrogenesis, regulation of cell signaling, matrix architecture, and new tissue formation [47,65]. Therefore, perlecan is a promising candidate molecule to investigate for a better understanding of cartilage repair mechanisms [66]. In adults, perlecan was found to be highly secreted during articular cartilage repair [48]. The role of perlecan in chondrogenesis and cartilage development indicates that it may have a potential function in repairing cartilage by reproducing its developmental roles in damaged or diseased tissues [66,67]. Perlecan plays a critical role in facilitating the maturation of chondroprogenitor stem cells and the creation of pluripotent migratory stem cell lineages, which affect joint formation and early cartilage development [68]. Therefore, heparan sulfate-deficient perlecan may exert inhibitory control over chondrocytes in mature cartilage, resulting in a poor healing response related to cartilage [66]. Curiously, in human knee OA cartilage, perlecan levels are significantly higher in areas close to cartilage defects [69]. Furthermore, collected data indicated that perlecan in the PCM of cartilaginous tissues is implicated in the regulation of biomechanical properties that characterize PCM´s various matrices, and may thus have unique applications in chondral regeneration [67].

### 4.4. Molecular Biomarkers

A biomarker is defined as “a characteristic that is objectively measured and evaluated as an indicator of normal biological processes, pathogenic processes, or pharmacologic responses to a therapeutic intervention” [70]. These molecules are mainly used to diagnose illness, predict illness, or assess a patient´s physical condition [70,71]. Biomarkers in chondral injuries can be extracted from serum, urine, or synovial fluid samples [72].

Although radiographs and other types of joint imaging are regularly used as diagnostic techniques for chondral injuries, they do not have the capacity to determine dynamic changes in the joint. Therefore, it is important to use molecular biomarkers, which, in addition to complementing biomedical imaging, allow for the monitoring of disease progression and the efficacy of treatment [58,73,74]. In chondral injuries, due to the fragmentation of specific matrix molecules, such as PGs, some of the fragments that are released can be analyzed. Over time, assays have been developed and successfully used to verify that PGs are indeed useful as biomarkers for monitoring the activity of the tissue destruction process [75].

Aggrecan is one of the most commonly studied cartilage proteins for biomarker development [76]. As previously reported, the proteolysis of aggrecan is an early feature of cartilage degradation following chondral injury. Therefore, the elevated presence of aggrecan fragments in synovial fluid is associated with joint injury and/or OA [77]. These fragments are measurable as an increase in the aggrecan released from the cartilage into the synovial fluid [78]. Aggrecan fragments present in synovial fluid can be detected via amino acid sequencing, Western blot, and enzyme-linked immunosorbent assay (ELISA) [77].

The release of soluble forms of biglycan or decorin from the ECM of cartilage into the synovial fluid following tissue injury may act as an internal danger signal [64]. Soluble biglycan and decorin in synovial fluid and serum can be detected using ELISA [63,64,78,79]. Barreto et al. [63] observed high levels of biglycan in advanced OA, concluding that soluble biglycan can serve as a mediator of OA cartilage as well as a potential biomarker. Other studies determined that increased serum decorin levels may indicate changes in ECM and are a risk factor associated with OA [79,80].

## 5. Analysis of Proteoglycans in Repaired Articular Cartilage

Focal cartilage defects are widespread, with up to 63% of the general population and 36% of athletes affected. In particular, larger defects can be challenging, particularly for individuals leading an active lifestyle [19,81]. These defects can lead to accelerated damage, increased pain, and even the progression of OA [82]. Given the high incidence and associated costs of chondral injuries, it is crucial to identify effective treatments. Finding the molecules involved in chondral repair processes could be key in the search for a treatment. 

A decreased PG concentration was shown to predispose cartilage tissue to micro-damage from mechanical loading, thereby weakening and altering the matrix´s structural integrity. Therefore, it is of vital importance to check the concentration and distribution of PGs in repaired articular cartilage [83], bearing in mind that in order to understand the properties of articular cartilage, it is necessary to appreciate the composition of the cartilage according to its layers (superficial, middle, deep, and calcified) and subregions (peri-cellular, territorial, and interterritorial) [84], as shown in Figure 2.

There are several animal and human studies in the literature that investigate the effectiveness of different treatment techniques for chondral and/or osteochondral defects. In human clinical trials and case studies, diagnostic imaging tests are commonly used since they are non-invasive examinations, whereas only a few studies reported the application of histological assessments. In animal experimental studies, histological evaluations have been commonly applied. However, not all these studies included in-depth histological analyses where molecules of great importance in the ECM, such as PGs, were analyzed.

PG analysis has been included in some publications as part of the evaluation processes and the PG content in repaired cartilage has been investigated using different methods. Some techniques, such as Safranin O/fast green staining [84,85,86,87,88,89,90,91,92,93,94], evaluate all PGs as a whole, while other techniques, such as immunohistochemistry [66,85] (Figure 2) and real-time PCR [86] (Table 2 and Table 3), allow for the evaluation of specific PGs.

## 6. Conclusions

Proper articular function depends significantly on the structural integrity and composition of the ECM [15], in which PGs play a major role [18,19]. Aggrecan is the basis of the viscoelastic properties of cartilage and is crucial in cell–ECM interactions. Biglycan and decorin stabilize the ECM by regulating cartilage integrity. Versican is a transient PG present in chondral differentiation and is replaced by aggrecan in the early stages of chondrocyte differentiation. Perlecan contributes to the mechanosensory and functional properties of cartilage and to the stabilization of the ECM. 

Due to the important role of PGs in articular cartilage, it is of vital importance to assess the content and distribution of PGs in terms of chondral injury and/or repair. Additionally, the assessment of PG molecules, such as aggrecan fragments, biglycan, and decorin, could be used as a joint degradation biomarker for OA in patients. Furthermore, in experimental studies, PG analysis is a useful determinant to evaluate the quality of repaired tissue after the application of different treatment strategies for chondral injuries.

## Figures and Tables

**Table 1:** PGs classification. Modified from Iozzo et al. [28].

Location	Classification	Eponym	Predominant GAG
INTRACELLULAR	Secretory granules	Serglycin	Hep
CELL SURFACE	Transmembrane	Syndecan, 1–4	HS
		NG2	CS
		Betaglycan	CS/HS
		Phosphacan	CS
	GPI-Anchored	Glypican, 1–6	HS
PERICELLULAR	Basement membrane zone	Perlecan	HS
		Agrin	HS
		Collagen XVIII	HS
		Collagen XV	CS/HS
EXTRACELLULAR	Hyalectan lectican	Aggrecan	CS/KS
		Versican	CS
		Neurocan	CS
		Brevican	CS
	Canonical	Biglycan	CS
		Decorin	DS
		Asporin	
		ECM2	
		ECMX	
		Fibromodulin	KS
		Lumican	KS
		PRELP	
		Keratocan	KS
		Osteoadherin	KS
		Epiphycan	DS/CS
		Opticin	
		Osteoglycin	
	Non-canonical	Chondroadherin	
		Nyctalopin	
		Tsukushi	
		Podocan	
		Podocan-Like 1	
	Spock	Testican, 1–3	HS

GAG: glycosaminoglycan; Hep: heparin; HS: heparan sulfate; CS: chondroitin sulfate; KS: keratan sulfate; DS: dermatan sulfate.

**Table 2:** Review of human clinical trials and case reports involving PGs analysis in repaired cartilage after different treatments in chondral osteochondral injuries.

Study	Species	Chondral Lesion Type	Reparative Treatment	Detection Technique	PGs Analyzed	Main Results
Garcia et al., 2021 [66]	Human	n/r	Autologous cell therapy	IHQ	Perlecan	Immunostaining for perlecan was significantly greater in autologous cell therapy repair tissues.
Levinson et al., 2019 [87]	Human	n/r	Minced cartilage	Safranin O/fast green	PGs altogether	Staining with Safranin O was positive, however, the outgrowth potential, the viability, and the matrix deposition were not different between the mincing techniques.
Hoffman et al., 2015 [88]	Human	Full-thickness chondral defect	Marrow stimulation with a viable chondral allograft	Safranin O/fast green	PGs altogether	The Safranin O staining revealed ample PG content throughout the majority of the tissue.

IHQ: immunohistochemical staining; n/r: not reported.

**Table 3:** Review of animal experimental studies involving PGs analysis in repaired cartilage after different treatments in chondral osteochondral injuries.

Study	Species	Chondral Lesion Type	Reparative Treatment	Detection Technique	PGs Analyzed	Main Results
Yan et al., 2020 [89]	Minipg	Full-thickness chondral defect	PRP combined with injectable HA hydrogel	Safranin O/fast green	PGs altogether	The HA hydrogel combined with PRP-treated group showed more hyaline-like cartilage with histological staining without formation of hypertrophic cartilage.
Passino et al., 2017 [90]	Ovine	PCD	Radioelectric asymmetric conveyor	Safranin O/fast green	PGs altogether	Histologically, the formation of immature hyaline articular cartilage was reported but with some slight irregularities and deformations on cartilage surface.
Pfeifer et al., 2017 [91]	Minipi	Full- and partial-thickness chondral defect	Microfracture	Safranin O/fast green	PGs altogether	Quantification of histology showed equal overall assessment for the FCD groups and better overall assessment in juvenile animals treated with microfracture.
Shi et al., 2016 [92]	Rabbit	Full-thickness chondral defect	Photo-cross-linked scaffold with kartogenin nanoparticles	Safranin O/fast green	PGs altogether	The regenerated tissues were close to the natural hyaline cartilage based on the histological test.
Christensen et al., 2015 [93]	Minipig	Full- and partial-thickness chondral defect	MACI, ADTT, microfracture, autologous bone graft, or autologous cartilage chips	Safranin O/fast green	PGs altogether	The histological results did not show complete regeneration of hyaline cartilage in any treatment group.
Peck et al., 2015 [94]	Minipig	n/r	LhCG	Safranin O/fast green and DMMB	PGs altogether	Microscopic inspection revealed that the living hyaline cartilaginous graft produces abundant cartilage-specific matrix molecules.
Mori et al., 2013 [86]	Rabbit	Full-thickness chondral defect	Implantation of the OP1-SCS	Safranin O/fast green and real-time PCR	PGs altogether and Aggrecan	Osteochondral defects treated with osteogenic protein 1. Salmon-derived collagen sponge disc showed cartilage tissue richness in PGs. Furthermore, histological assessment indicated that the score was significantly greater.
Pretzel et al., 2013 [64]	Bovine	Full-thickness chondral defect	Self-healing of resident cartilage cells in conjunction with bacterial nanocellulose	Safranin O/fast green and IHQ	PGs altogether and Aggrecan	Chondrocytes on the bacterial nanocellulose showed signs of successful redifferentation over time, including an increase in aggrecan.
Milán et al., 2010 [95]	Ovine	Full-thickness chondral defect	PRP combined with MFx	Safranin O/fast green	PGs altogether	Histological analysis revealed that none of the experimental treatments produced hyaline cartilage.

HA: hyaluronic acid; n/r: not reported; ADTT: autologous-dual-tissue transplantation; DMMB: 1,9-dimethylmethylene blue; MACI: matrix-induced autologous chondrocyte implantation; PCR: polymerase chain reaction; PRP: platelet-rich plasma.

## Data Availability

The data presented in this study are available on request from the corresponding author.

## Abbreviations

ADTT	Autologous-dual-tissue transplantation
CSPG	Chondroitin sulfate proteoglycan
DMMB	1,9-dimethylmethylene blue
DSPG	Dermatan sulfate proteoglycan
ECM	Extracellular matrix
ELISA	Enzyme-linked immunosorbent assay
GAG	Glycosaminoglycan
GPI	Glycosyl-phosphatidyl-inositol
HA	Hyaluronic acid
Hep	Heparan
HSPG	Heparan sulfate proteoglycan
IHQ	Immunohistochemical staining
KSPG	Keratan sulfate proteoglycan
MACI	Matrix-induced autologous chondrocyte implantation
n/r	Not reported
OA	Osteoarthritis
PCM	Pericellular matrix
PCR	Polymerase chain reaction
PG	Proteoglycan
PRP	Platelet-rich plasma
SLRP	Small leucine-rich proteoglycan

## References

[1] Piez K.A. (1997). History of extracellular matrix: A personal view. Matrix Biol..

[2] Theocharis A.D., Skandalis S.S., Gialeli C., Karamanos N.K. (2016). Extracellular matrix structure. Adv. Drug Deliv. Rev..

[3] Yue B. (2014). Biology of the extracellular matrix: An overview. J. Glaucoma..

[4] Hastings J.F., Skhinas J.M., Fey D., Croucher D.R., Cox T.R. (2019). The extracellular matrix as a key regulator of intracellular signalling networks. Br. J. Pharmacol..

[5] Jurj A., Ionescu C., Berindan-Neagoe I., Braicu C. (2022). The extracellular matrix alteration, implication in modulation of drug resistance mechanism: Friends or foes?. J. Exp. Clin. Cancer Res..

[6] Han B., Li Q., Wang C., Patel P., Adams S.M., Doyran B., Nia H.T., Oftadeh R., Zhou S., Li C.Y. (2019). Decorin regulates the aggrecan network integrity and biomechanical functions of cartilage extracellular matrix. ACS Nano.

[7] Gilbert S.J., Bonnet C.S., Blain E.J. (2021). Mechanical Cues: Bidirectional Reciprocity in the Extracellular Matrix Drives Mechano-Signalling in Articular Cartilage. Int. J. Mol. Sci..

[8] Alcaide-Ruggiero L., Molina-Hernández V., Granados M.M., Domínguez J.M. (2021). Main and Minor Types of Collagens in the Articular Cartilage: The Role of Collagens in Repair Tissue Evaluation in Chondral Defects. Int. J. Mol. Sci..

[9] Xia Y., Darling E.M., Herzog W. (2018). Functional Properties of Chondrocytes and Articular Cartilage using Optical Imaging to Scanning Probe Microscopy. J. Orthop. Res..

[10] Larson C.M., Kelley S.S., Blackwood A.D., Banes A.J., Lee G.M. (2002). Retention of the native chondrocyte pericellular matrix results in significantly improved matrix production. Matrix Biol..

[11] Wiberg C., Klatt A.R., Wagener R., Paulsson M., Bateman J.F., Heinegård D., Mörgelin M. (2003). Complexes of matrilin-1 and biglycan or decorin connect collagen VI microfibrils to both collagen II and aggrecan. J. Biol. Chem..

[12] Ulrich-Vinther M., Maloney M.D., Schwarz E.M., Rosier R., O’Keefe R.J. (2003). Articular cartilage biology. J. Am. Acad. Orthop. Surg..

[13] Bhosale A.M., Richardson J.B. (2008). Articular cartilage: Structure, injuries and review of management. Br. Med. Bull..

[14] Simon T.M., Jackson D.W. (2006). Articular cartilage: Injury pathways and treatment options. Sport. Med. Arthrosc. Rev..

[15] Huey D.J., Hu J.C., Athanasiou K.A. (2012). Unlike bone, cartilage regeneration remains elusive. Science.

[16] Roughley P.J., Mort J.S. (2014). The role of aggrecan in normal and osteoarthritic cartilage. J. Exp. Orthop..

[17] Prakash D., Learmonth D. (2002). Natural progression of osteo-chondral defect in the femoral condyle. Knee.

[18] Helmick C.G., Felson D.T., Lawrence R.C., Gabriel S., Hirsch R., Kwoh C.K., Liang M.H., Kremers H.M., Mayes M.D., Merkel P.A. (2008). Estimates of the prevalence of arthritis and other rheumatic conditions in the United States. Part I. Arthritis Rheum..

[19] Alberton P., Dugonitsch H.C., Hartmann B., Li P., Farkas Z., Saller M.M., Clausen-Schaumann H., Aszodi A. (2019). Aggrecan hypomorphism compromises articular cartilage biomechanical properties and is associated with increased incidence of spontaneous osteoarthritis. Int. J. Mol. Sci..

[20] Krishnan Y., Grodzinsky A.J. (2018). Cartilage diseases. Matrix Biol..

[21] Martin J.A., Buckwalter J.A. (2001). Roles of Articular Cartilage Aging and Chondrocyte Senescence in the Pathogenesis of Osteoarthritis. Iowa Orthop. J..

[22] Masson A.O., Krawetz R.J. (2020). Understanding cartilage protection in OA and injury: A spectrum of possibilities. BMC Musculoskelet. Disord..

[23] Vynios D.H. (2014). Metabolism of cartilage proteoglycans in health and disease. Biomed. Res. Int..

[24] Lord M.S., Whitelock J.M. (2013). Recombinant production of proteoglycans and their bioactive domains. FEBS J..

[25] Couchman J.R., Pataki C.A. (2012). An introduction to proteoglycans and their localization. J. Histochem. Cytochem..

[26] Sajdera S.W., Hascall V.C. (1969). Proteinpolysaccharide Complex from bovine nasal cartilage: A comparison of low and high shear extraction procedures. J. Biol. Chem..

[27] Iozzo R.V., Schaefer L. (2015). Proteoglycan form and function: A comprehensive nomenclature of proteoglycans. Matrix Biol..

[28] Douaiher J., Succar J., Lancerotto L., Gurish M.F., Orgill D.P., Hamilton M.J., Krilis S.A., Stevens R.L. (2014). Development of mast cells and importance of their tryptase and chymase serine proteases in inflammation and wound healing. Adv. Immunol..

[29] Korpetinou A., Skandalis S.S., Labropoulou V.T., Smirlaki G., Noulas A., Karamanos N.K., Theocharis A.D. (2014). Serglycin: At the crossroad of inflammation and malignancy. Front. Oncol..

[30] Kolset S.O., Pejler G. (2011). Serglycin: A structural and functional chameleon with wide impact on immune cells. J. Immunol..

[31] Matsumoto K., Kamiya N., Suwan K., Atsumi F., Shimizu K., Shinomura T., Yamada Y., Kimata K., Watanabe H. (2006). Identification and characterization of versican/PG-M aggregates in cartilage. J Biol. Chem..

[32] Aspberg A. (2012). The different roles of aggrecan interaction domains. J. Histochem. Cytochem..

[33] Hascall V.C., Heinegård D. (1974). Aggregation of Cartilage Proteoglycans: II. Oligosaccharide competitors of the proteoglycan-hyaluronic acid interaction. J. Biol. Chem..

[34] Mouw J.K., Ou G., Weaver V.M. (2014). Extracellular matrix assembly: A multiscale deconstruction. Nat. Rev. Mol. Cell Biol..

[35] Hayes A.J., Melrose J. (2020). Aggrecan, the primary weight-bearing cartilage proteoglycan, has context-dependent, cell-directive properties in embryonic development and neurogenesis: Aggrecan glycan side chain modifications convey interactive biodiversity. Biomolecules.

[36] Alcaide-Ruggiero L., Molina-Hernández V., Morgaz J., Fernández-Sarmiento J.A., Granados M.M., Navarrete-Calvo R., Pérez J., Quirós-Carmona S., Carrillo J.M., Cugat R. (2023). Particulate cartilage and platelet-rich plasma treatment for knee chondral defects in sheep. Knee Surg. Sport. Traumatol. Arthrosc..

[37] Alcaide-Ruggiero L., Molina-Hernández V., Morgaz J., Fernández-Sarmiento J.A., Granados M.M., Carrillo J.M., Cugat R., Domínguez J.M. Reparación de defectos condrales de rodilla en ovejas tras el tratamiento con cartílago autólogo particulado y plasma rico en plaquetas. Proceedings of the XXVII Congreso Internacional de la Sociedad Española de Cirugía Veterinaria.

[38] Neill T., Schaefer L., Iozzo R.V. (2012). Decorin: A Guardian from the Matrix. Am. J. Pathol..

[39] Nastase M.V., Young M.F., Schaefer L. (2012). Biglycan: A Multivalent Proteoglycan Providing Structure and Signals. J. Histochem. Cytochem..

[40] Han B., Li Q., Wang C., Chandrasekaran P., Zhou Y., Qin L., Liu X.S., Enomoto-Iwamoto M., Kong D., Iozzo R.V. (2021). Differentiated activities of decorin and biglycan in the progression of post-traumatic osteoarthritis. Osteoarthr. Cartil..

[41] Kram V., Shainer R., Jani P., Meester J.A.N., Loeys B., Young M.F. (2020). Biglycan in the skeleton. J. Histochem. Cytochem..

[42] Roughley P.J. (2001). Articular cartilage and changes in arthritis noncollagenous proteins and proteoglycans in the extracellular matrix of cartilage. Arthritis Res..

[43] Islam S., Watanabe H. (2020). Versican: A dynamic regulator of the extracellular matrix. J. Histochem. Cytochem..

[44] Choocheep K., Hatano S., Takagi H., Watanabe H., Kimata K., Kongtawelert P., Watanabe H. (2010). Versican facilitates chondrocyte differentiation and regulates joint morphogenesis. J. Biol. Chem..

[45] Smith S.M., Melrose J. (2019). Type XI collagen-perlecan-HS interactions stabilise the pericellular matrix of annulus fibrosus cells and chondrocytes providing matrix stabilisation and homeostasis. J. Mol. Hist..

[46] Whitelock J.M., Melrose J., Iozzo R.V. (2008). Diverse cell signaling events modulated by perlecan. Biochemistry.

[47] Hayes A.J., Farrugia B.L., Biose I.J., Bix G.J., Melrose J. (2022). Perlecan, A multi-functional, cell-instructive, matrix-stabilizing proteoglycan with roles in tissue development has relevance to connective tissue repair and regeneration. Front. Cell Dev. Biol..

[48] Guilak F., Hayes A.J., Melrose J. (2021). Perlecan in pericellular mechanosensory cell-matrix communication, extracellular matrix stabilisation and mechanoregulation of load-bearing connective tissues. Int. J. Mol. Sci..

[49] Hayes A.J., Hughes C.E., Smith S.M., Caterson B., Little C.B., Melrose J. (2016). The CS Sulfation Motifs 4C3, 7D4, 3B3[−]; and perlecan identify stem cell populations and their niches, activated progenitor cells and transitional areas of tissue development in the fetal human elbow. Stem Cells Dev..

[50] Heinegård D., Saxne T. (2011). The role of the cartilage matrix in osteoarthritis. Nat. Rev. Rheumatol..

[51] Poole C.A., Flint M.H., Beaumont B.W. (1987). Chondrons in cartilage: Ultrastructural analysis of the pericellular microenvironment in adult human articular cartilages. J. Orthop. Res..

[52] Twomey J.D., Thakore P.I., Hartman D.A., Myers E.G.H., Hsieh A.H. (2014). Roles of type VI collagen and decorin in human mesenchymal stem cell biophysics during chondrogenic differentiation. Eur. Cells Mater..

[53] Hayes A.J., Shu C.C., Lord M.S., Little C.B., Whitelock J.M., Melrose J. (2016). Pericellular colocalisation and interactive properties of type VI collagen and perlecan in the intervertebral disc. Eur. Cells Mater..

[54] Suutre S., Kerna I., Lintrop M., Tamm H., Aunapuu M., Arend A., Tamm A. (2015). Evaluation of correlation of articular cartilage staining for DDR2 and proteoglycans with histological tissue damage and the results of radiographic assessment in patients with early stages of knee osteoarthritis. Int. J. Clin. Exp. Pathol..

[55] Lu X.L., Mow V.C., Guo X.E. (2009). Proteoglycans and mechanical behavior of condylar cartilage. J. Dent. Res..

[56] Maroudas A., Ziv I., Weisman N., Venn M. (1985). Studies of hydration and swelling pressure in normal and osteoarthritic cartilage. Biorheology.

[57] Sandy J.D., Verscharen C. (2001). Analysis of aggrecan in human knee cartilage and synovial fluid indicates that aggrecanase (ADAMTS) activity is responsible for the catabolic turnover and loss of whole aggrecan whereas other protease activity is required for C-terminal processing in vivo. Biochem. J..

[58] Kumavat R., Kumar V., Malhotra R., Pandit H., Jones E., Ponchel F., Biswas S. (2021). Biomarkers of Joint Damage in Osteoarthritis: Current Status and Future Directions. Mediat. Inflamm..

[59] Larsson S., Englund M., Struglics A., Lohmander L.S. (2010). Association between synovial fluid levels of aggrecan ARGS fragments and radiographic progression in knee osteoarthritis. Arthritis Res. Ther..

[60] Poole A.R., Rosenberg L.C., Reiner A., Ionescu M., Bogoch E., Roughley P.J. (1996). Contents and distributions of the proteoglycans decorin and biglycan in normal and osteoarthritic human articular cartilage. J. Orthop. Res..

[61] Ni G.X., Li Z., Zhou Y.Z. (2014). The role of small leucine-rich proteoglycans in osteoarthritis pathogenesis. Osteoarthr. Cartil..

[62] Gronau T., Krüger K., Prein C., Aszodi A., Gronau I., Iozzo R.V., Mooren F.C., Clausen-Schaumann H., Bertrand J., Pap T. (2017). Forced exercise-induced osteoarthritis is attenuated in mice lacking the small leucine-rich proteoglycan decorin. Ann. Rheum. Dis..

[63] Barreto G., Soininen A., Ylinen P., Sandelin J., Konttinen Y.T., Nordström D.C., Eklund K.K. (2015). Soluble biglycan: A potential mediator of cartilage degradation in osteoarthritis. Arthritis Res. Ther..

[64] Anders H.J., Schaefer L. (2014). beyond tissue injury—Damage-associated molecular patterns, toll-like receptors, and inflammasomes also drive regeneration and fibrosis. J. Am. Soc. Nephrol..

[65] Gomes R.R., Farach-Carson M.C., Carson D.D. (2004). Perlecan functions in chondrogenesis: Insights from in vitro and in vivo models. Cells Tissues Organs..

[66] Garcia J., McCarthy H.S., Kuiper J.H., Melrose J., Roberts S. (2021). Perlecan in the natural and cell therapy repair of human adult articular cartilage: Can modifications in this proteoglycan be a novel therapeutic approach?. Biomolecules.

[67] Gao G., Chen S., Pei Y.A., Pei M. (2021). Impact of perlecan, a core component of basement membrane, on regeneration of cartilaginous tissues. Acta Biomater..

[68] Melrose J., Hayes A.J., Whitelock J.M., Little C.B. (2008). Perlecan, the “jack of all trades” proteoglycan of cartilaginous weight-bearing connective tissues. BioEssays.

[69] Tesche F., Miosge N. (2004). Perlecan in late stages of osteoarthritis of the human knee joint. Osteoarthr. Cartil..

[70] Biomarkers Definitions Working Group (2001). Biomarkers and surrogate endpoints: Preferred definitions and conceptual framework. Clin. Pharmacol. Ther..

[71] Mobasheri A., Henrotin Y. (2015). Biomarkers of (osteo)arthritis. Biomarkers.

[72] Boffa A., Merli G., Andriolo L., Lattermann C., Salzmann G.M., Filardo G. (2021). Synovial Fluid Biomarkers in Knee Osteoarthritis: A Systematic Reviewand Quantitative Evaluation Using BIPEDs Criteria. Cartilage.

[73] Braun H.J., Gold G.E. (2012). Diagnosis of Osteoarthritis: Imaging. Bone.

[74] Marouf B.H. (2021). Effect of Resveratrol on Serum Levels of Type II Collagen and Aggrecan in Patients with Knee Osteoarthritis: A Pilot Clinical Study. BioMed Res. Int..

[75] Heinegård D. (2009). Proteoglycans and more-from molecules to biology. Int. J. Exp. Pathol..

[76] Bay-Jensen A.C., Mobasheri A., Thudium C.S., Kraus V.B., Karsdal M.A. (2022). Blood and urine biomarkers in osteoarthritis—An update on cartilage associated type II collagen and aggrecan markers. Curr. Opin. Rheumatol..

[77] Larsson S., Lohmander L.S., Struglics A. (2009). Synovial fluid level of aggrecan ARGS fragments is a more sensitive marker of joint disease than glycosaminoglycan or aggrecan levels: A cross-sectional study. Arthritis Res. Ther..

[78] Lohmander L.S., Dahlberg L., Ryd L., Heinegård D. (1989). Increased levels of proteoglycan fragments in knee joint fluid after injury. Arthritis Rheum..

[79] Ozler K. (2021). Relationship between increased serum & synovial fluid decorin levels & knee osteoarthritis. Indian J. Med. Res..

[80] Anwar R., Habib B., Mustafa Y., Kazi A., Farooq O., Nishat M. (2022). Validation of Levels of Decorin as A Reliable Biomarker of Osteoarthritis: Comparison of Serum and Synovial Fluid Levels. Pak. J. Med. Health Sci..

[81] Perera J.R., Gikas P.D., Bentley G. (2012). The present state of treatments for articular cartilage defects in the knee. Ann. R. Coll. Surg. Engl..

[82] Everhart J.S., Boggs Z., DiBartola A.C., Wright B., Flanigan D.C. (2021). Knee Cartilage Defect Characteristics Vary among SymptomaticRecreational and Competitive Scholastic Athletes Eligible for Cartilage Restoration Surgery. Cartilage.

[83] Pastrama M.I., Ortiz A.C., Zevenbergen L., Famaey N., Gsell W., Neu C.P., Himmelreich U., Jonkers I. (2019). Combined enzymatic degradation of proteoglycans and collagen significantly alters intratissue strains in articular cartilage during cyclic compression. J. Mech. Behav. Biomed. Mater..

[84] Hsueh M.F., Khabut A., Kjellström S., Önnerfjord P., Kraus V.B. (2016). Elucidating the Molecular Composition of Cartilage by Proteomics. J. Proteome Res..

[85] Pretzel D., Linss S., Ahrem H., Endres M., Kaps C., Klemm D., Kinne R.W. (2013). A novel in vitro bovine cartilage punch model for assessing the regeneration of focal cartilage defects with biocompatible bacterial nanocellulose. Arthritis Res. Ther..

[86] Mori H., Kondo E., Kawaguchi Y., Kitamura N., Nagai N., Iida H., Yasuda K. (2013). Development of a salmon-derived crosslinked atelocollagen sponge disc containing osteogenic protein-1 for articular cartilage regeneration: In vivo evaluations with rabbits. BMC Musculoskelet. Disord..

[87] Yan W., Xu X., Xu Q., Sun Z., Jiang Q., Shi D. (2019). Platelet-rich plasma combined with injectable hyaluronic acid hydrogel for porcine cartilage regeneration: A 6-month follow-up. Regen. Biomater..

[88] Levinson C., Cavalli E., Sindi D.M., Kessel B., Zenobi-Wong M., Preiss S., Salzmann G., Neidenbach P. (2019). Chondrocytes From device-minced articular cartilage show potent outgrowth into fibrin and collagen hydrogels. Orthop. J. Sport. Med..

[89] Passino E.S., Rocca S., Caggiu S., Columbano N., Castagna A., Fontani V., Rinaldi S. (2017). REAC regenerative treatment efficacy in experimental chondral lesions: A pilot study on ovine animal model. Clin. Interv. Aging.

[90] Pfeifer C.G., Fisher M.B., Saxena V., Kim M., Henning E.A., Steinberg D.A., Dodge G.R., Mauck R.L. (2017). Age-dependent subchondral bone remodeling and cartilage repair in a minipig defect model. Tissue Eng. Part C Methods.

[91] Shi D., Xu X., Ye Y., Song K., Cheng Y., Di J., Hu Q., Li J., Ju H., Jiang Q. (2016). Photo-cross-linked scaffold with kartogenin-encapsulated nanoparticles for cartilage regeneration. ACS Nano.

[92] Hoffman J.K., Geraghty S., Protzman N.M. (2015). Articular Cartilage Repair Using Marrow Stimulation Augmented with a Viable Chondral Allograft: 9-Month Postoperative Histological Evaluation. Case Rep. Orthop..

[93] Christensen B.B., Foldager C.B., Olesen M.L., Vingtoft L., Rölfing J.H.D., Ringgaard S., Lind M. (2015). Experimental articular cartilage repair in the Göttingen minipig: The influence of multiple defects per knee. J. Exp. Orthop..

[94] Peck Y., He P., Chilla G.S.V.N., Poh C.L., Wang D.A. (2015). A preclinical evaluation of an autologous living hyaline-like cartilaginous graft for articular cartilage repair: A pilot study. Sci. Rep..

[95] Milano G., Sanna-Passino E., Deriu L., Careddu G., Manunta L., Manunta A., Saccomanno M.F., Fabbriciani C. (2010). The effect of platelet rich plasma combined with microfractures on the treatment of chondral defects: An experimental study in a sheep model. Osteoarthr. Cartil..

